# Accelerated RAKI reconstruction for multi‐slice cardiac cine applications

**DOI:** 10.1002/mp.70145

**Published:** 2025-11-19

**Authors:** Lucile Quillien, Julien Oster, Pierre‐André Vuissoz

**Affiliations:** ^1^ IADI, INSERM U1254 Université de Lorraine Nancy France; ^2^ CIC‐IT, INSERM 1433 Université de Lorraine, CHRU Nancy Nancy France

**Keywords:** cardiac 2D cine, deep‐learning MRI reconstruction, parallel imaging

## Abstract

**Background:**

Accelerated MRI reconstruction techniques are necessary to avoid long cardiac exams. K‐space‐based parallel imaging (PI) reconstruction has recently been adapted to deep learning with a scan‐specific training technique entitled scan‐specific robust artificial neural‐networks for k‐space interpolation (RAKI), which incorporates nonlinearity by applying convolutional neural networks. While the scan‐specific aspect alleviates the need for a large training database, as it consists of a single‐shot training, it consequently increases the overall reconstruction time.

**Purpose:**

The aim of this study is to adapt RAKI reconstruction to cardiac cine acquisitions by optimizing the training strategy and exploiting the spatio‐temporal redundancy while ensuring image quality.

**Methods:**

Ten fully sampled multi‐slice cine data from the public cardiac OCMR database were used to compare the proposed method to standard reconstruction techniques (GRAPPA, RAKI, and rRAKI). To accelerate the reconstruction, the RAKI algorithm was simplified by removing the nonlinear activation units and reducing the number of layers, making it a parallelized GRAPPA‐like reconstruction with only one convolution layer. Training of the weights was further accelerated by training only specific slices and cardiac phases of the whole cine stack of images. Image quality metrics such as the PSNR, NMSE, and SSIM were computed to evaluate the image quality, while the reconstruction time was also assessed.

**Results:**

Quality metrics showed comparable results to state‐of‐the‐art methods, while the average reconstruction time was reduced by 40 on average compared to GRAPPA, RAKI, and rRAKI.

**Conclusions:**

The reconstructions for the proposed method showed comparable image quality to standard methods while being significantly faster. Some “striping” artifacts remain with our method, which seem to be directly linked to the k‐space‐based optimization process.

## 1 INTRODUCTION

Cardiac MRI (CMR) is a widely used imaging modality, which has become the reference clinical examination for many cardiomyopathy diagnoses.^1^ Long acquisition time is one of the major drawbacks of MRI, with CMR being a particular case with motion management requiring cardiac synchronization and repetitive breath‐holds. Reducing the acquisition time while maintaining imaging quality is therefore a very important topic of research in the domain.

One of the most common acceleration techniques widely adopted in clinical routine is parallel imaging (PI), which uses multiple receiver coils to simultaneously acquire raw data, allowing the reduction of acquired k‐space lines.^2^ These different signals can then be combined to get a final image of good imaging quality, even when undersampled with different acceleration rates (R). However, the noise amplification is often too critical to ensure sufficient imaging quality and clinical diagnosis when going beyond an acceleration rate of 4.^3^ PI techniques can be separated into two main classes of methods: (i) sensitivity encoding (SENSE)^4^ and (ii) generalized autocalibrating partially parallel acquisitions (GRAPPA),^5^ both being currently available on most MRI scanners. SENSE operates in the image domain and uses coil sensitivity information to solve a linear inverse problem and obtain the final unaliased image, while the interpolation of the missing lines is performed directly in k‐space for GRAPPA.[Supplementary-material mp70145-supl-0001]

Another very famous and widely used reconstruction method is compressed sensing (CS),^6^ which offers an even higher acceleration factor, especially in dynamic settings such as cardiac MRI.^7^ CS can go beyond the Nyquist theorem by taking advantage of a sparse representation of MRI images, randomly undersampling the raw data, and using L1 regularization during reconstruction to recover the highly undersampled image.

Deep learning (DL) techniques have become increasingly popular in the MR image reconstruction field as they offer the possibility of large acceleration rates (R>4)^8^ by learning a compact latent representation of the MRI images, which therefore requires access to large databases with raw data and high‐quality reconstructed MRI. Due to the earlier availability of such large databases for brain and knee MRI, DL‐based MRI reconstruction techniques have initially been applied to knee or brain images. However, cardiac MRI reconstruction applications have recently been proposed thanks to the release of a few databases^9^ or challenges.^10^ Current DL approaches for cardiac MRI reconstruction can be divided into three categories: (i) post‐processing techniques directly learning an end‐to‐end mapping between undersampled k‐space data and ground‐truth reconstructed images with a neural net; (ii) model‐driven unrolled methods consisting of iteratively denoising images with a learnable neural net followed by a data consistency step ensuring the simulated k‐space from the reconstructed image stays close to the acquired raw data; and (iii) finally scan‐specific k‐space‐based interpolation techniques. The first two categories require being trained on a “large” set of data in order to learn an optimal set of parameters. Given the size of available databases (few hundred subjects with tens of pairs of k‐spaces and images), it is quite likely that these solutions might have a tendency to introduce a bias toward healthy (or most common) cardiac MRI representations and might therefore not be robust against domain shifts or overfitting. Whereas the latter category relies on a one‐shot learning strategy, therefore requiring full training for each new subject. Such an approach ensures that the proposed reconstructed images will not be biased toward a healthy (or a given) population. However, since parameters are only learned from a single patient, lower acceleration rates can be achieved. In the following of this study, we will focus solely on the use of these k‐space interpolation techniques. Scan‐specific robust artificial neural networks for k‐space interpolation (RAKI)^11^ were proposed to alleviate the need for such a large database, by extending GRAPPA with the addition of nonlinear activation layers and further convolutional layers. Training of the convolutional neural network weights (CNN) is performed using only the patient ACS data, making it a single‐shot DL technique and therefore quite attractive. RAKI has since been extended to simultaneous multi‐slice data.^12^, ^13^, ^14^ An alternative version has recently been proposed, denoted residual RAKI^15^ (rRAKI), that combines the initial RAKI network with a parallel network consisting of a single linear layer. rRAKI was shown to reduce residual artifacts on reconstructed image quality by guiding the weight learning to physics‐based solutions and adding interpretability. The main limitation of both RAKI and rRAKI lies in the need for a scan‐specific training of the model, which increases the overall reconstruction time. Furthermore, since scan‐specific models are single‐shot, high acceleration rates are more difficult to achieve than conventional methods relying on deep image priors trained on huge databases. However, they should instead be less biased toward normal morphologies and less sensitive to hallucinations while preserving anomalies in the reconstructed image.

The purpose of this study is to determine the possibility of accelerating RAKI‐based reconstruction specifically for a cardiac cine acquisition. We will try to assess whether it is possible to further accelerate the reconstruction process while guaranteeing imaging quality by using the spatio‐temporal redundancy for multi‐slice cine acquisitions and by comparing different RAKI model architectures (especially evaluating the need for additional nonlinear layers introduced in RAKI).

## 2 METHODS

In this paper, a lighter and simplified RAKI architecture is proposed for a cardiac cine application. Moreover, a training strategy to further accelerate the reconstruction process by exploiting the information redundancy between cardiac phases and adjacent slices is proposed. Fully sampled multi‐slice cine data from the public cardiac OCMR database^9^ was used to assess this new architecture. The proposed model was compared to state‐of‐the‐art techniques, including GRAPPA, RAKI, and residual RAKI, in terms of image quality and reconstruction time. Finally, an ablation study was proposed to assess the contribution of each proposed architecture choice.

### 2.1 State‐of‐the‐art techniques

**FIGURE 1:**
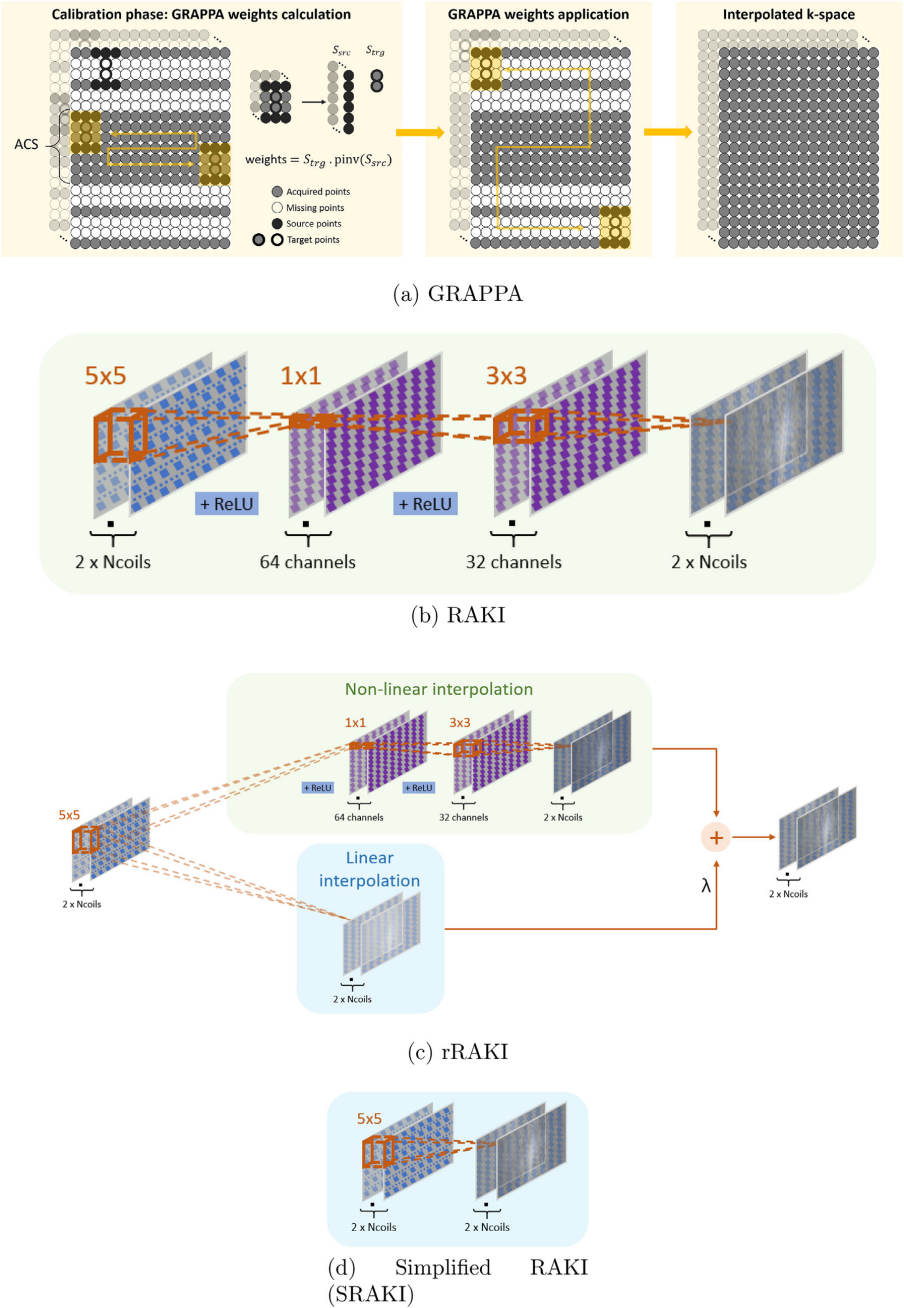
GRAPPA reconstruction procedure (a) and network architectures for (b) RAKI, (c) rRAKI, and (d) SRAKI implementations, with kernel sizes and the position of the rectified linear units (ReLU) activation functions. The number of parameters for data acquired with 15 coils is (b) 58 814, (c) 81 344, and (d) 22 530.

GRAPPA works in two steps: (i) during calibration, the interpolating convolution kernel is estimated using the auto‐calibrated signal (ACS), which comprises the fully sampled k‐space central lines. Multiple observations are then possible depending on the size of the ACS and the undersampling rate (Figure 1a). GRAPPA weights can then be estimated from these observations by solving the Moore–Penrose pseudoinverse using the least squares method. (ii) The estimated kernel is then applied to the entire k‐space, and the missing lines are interpolated to fill the k‐space. Finally, a Fourier transform with coil combination, usually using a sum of squares (SoS), is performed to reconstruct the image.

Compared to GRAPPA, RAKI introduced additional nonlinear activation layers and two additional CNN layers. As for GRAPPA, this technique requires the acquisition of fully sampled central lines (ACS). The CNN weights can then be trained using classical DL gradient descent on the data provided for this specific scan, making it really appealing, as it does not require access to a large database. RAKI was implemented in PyTorch using the online available library (https://github.com/geopi1/DeepMRI). Minor modifications were made to obtain the architecture presented in Figure 1b. The final RAKI model contains three convolution layers (with kernel sizes of [5 × 5], [1 × 1], and [3 × 3]), the first two layers being followed by a rectified linear unit (ReLU) nonlinear activation layer. The complex k‐space data was split into imaginary and real parts, resulting in $2\times N_{coils}$ channels. The output of the network comprises the same number of coils ($2\times N_{coils}$), consisting of the full k‐space. The architecture tested in this study used a different number of channels (notably for the last layer) and different kernel sizes from the original architecture.^11^

The recently proposed residual RAKI (rRAKI)^15^ adds a second linear path, a simple linear CNN layer similar to GRAPPA, to the standard nonlinear RAKI network. The modified architecture is depicted in Figure 1c. The model is inspired by the architecture of a ResNet, the only difference being that the skip connection consists of a simple linear CNN instead of the usual identity function or [1 × 1] kernel. The linear path can be seen as an equivalent GRAPPA model, that helps guiding the training of the nonlinear path and serves as a regularization. This training is performed by optimizing a combined loss function, with the combination λ parameter set to λ=1.^15^

### 2.2 Cardiac 2D cine data acquisition

Cardiac 2D cine data are typically acquired during breath‐holds and synchronized with the heart contraction by detecting the R peak in the ECG signal.^1^ With a very short TR, multiple k‐space lines can be acquired in a single heartbeat, thus at different stages of the cardiac cycle. The acquisition is performed over multiple heartbeats until all k‐space lines are acquired for each cardiac phase. Data is then retrospectively reordered to obtain multiple k‐spaces of different cardiac phases.

With the RAKI algorithm, each k‐space for all cardiac phases need to be trained independently, thus each requiring a specific ACS. As such, RAKI has a long reconstruction time due to its scan‐specific training. The idea in this study is to extend RAKI for cardiac cine application and accelerate its reconstruction pipeline by reducing the amount of required training. One can indeed assume that coil sensitivity maps remain fairly constant throughout the cardiac cycle. Similarly, although coil sensitivities are slice dependent, those maps should remain relatively stable on adjacent slices. The training of the network can therefore be limited to a subset of ACS from given cardiac phases and slices.

The proposed technique was assessed and compared to state‐of‐the‐art techniques on the publicly available OCMR database^9^ (available at https://ocmr.info/), comprising of cardiac cine data from both patients and volunteers. This study focused only on fully sampled multi‐slice data, which consisted of 10 stacks of images, with 10 to 14 slices. These images were acquired on different Siemens scanners (8 acquired at 1.5T and 2 at 3T) in short‐axis view. Imaging parameters were as follows (min/max): FOV = 600 × 233 mm^2^ / 760 × 308 mm^2^, matrix size=288 × 112/384 × 156, slice thickness=6 mm/8 mm, TR=2.81 ms/3.05 ms, TE=1.41 ms /1.5 3ms, flip angle=33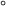/70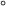, cardiac phases=18/0. For comparison purposes, only nine central adjacent slices were randomly selected for the reconstruction.

To assess the reconstruction time between all methods as well as the reconstruction quality, images were retrospectively undersampled with different uniform undersampling rates and numbers of ACS training lines: R=4 (e.g., keeping one in four k‐space line) with 26 ACS lines, R=5 with ACS = 30, and R=6 with ACS = 36.

### 2.3 Proposed method and ablation study

**FIGURE 2:**
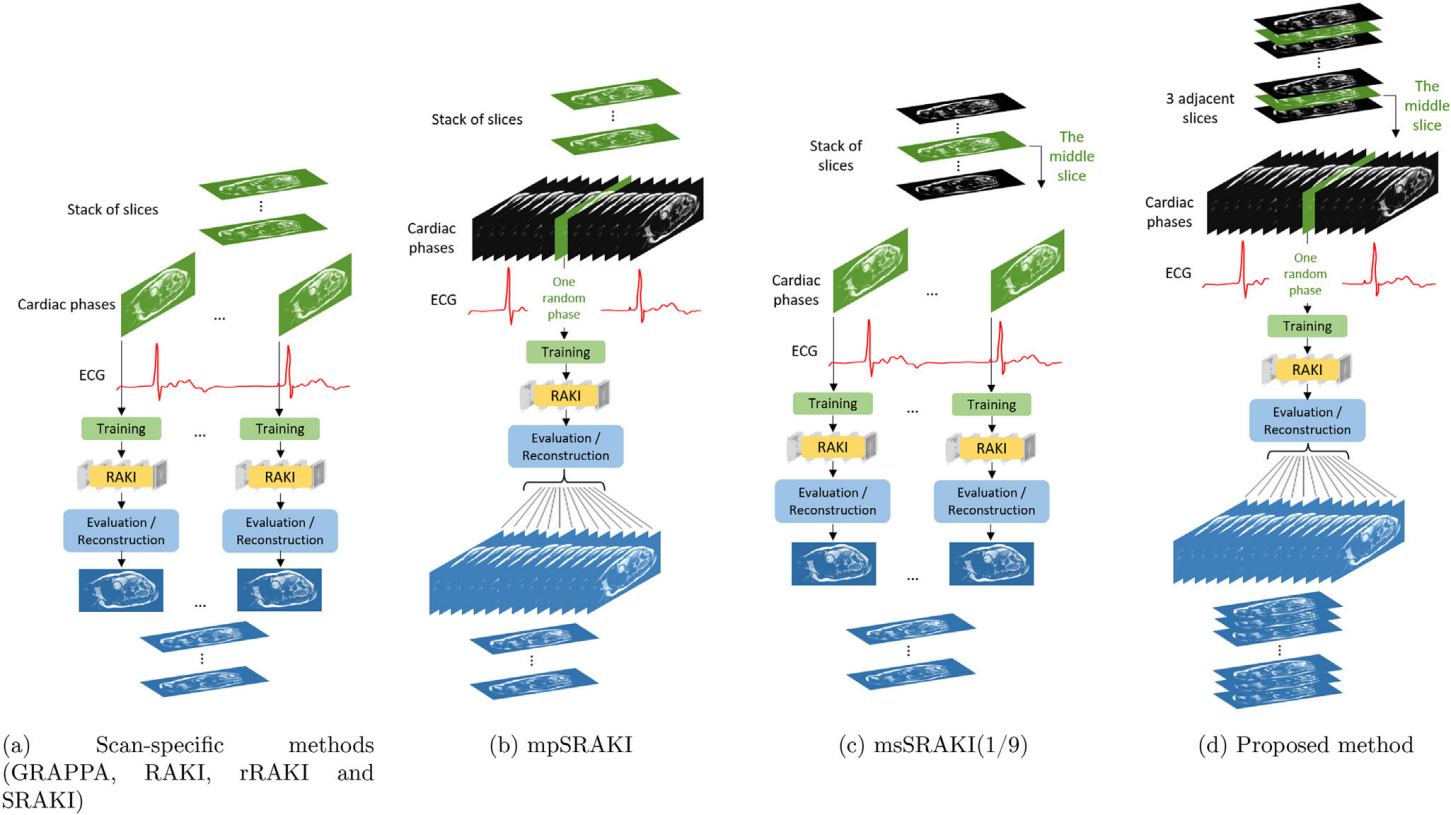
Flowchart of the different reconstruction methods for multi‐slice cine data: (a) Scan‐specific methods, (b) mpSRAKI, (c) msSRAKI(1/9), and (d) the proposed method. Scan‐specific methods comprise GRAPPA, RAKI, rRAKI, and SRAKI. For GRAPPA, the training part refers to the calibration phase with the estimation of the weights while the evaluation represents the application of the convolution kernel in k‐space. Images in green correspond to the “trained” images, while black images are not trained by the network. Blue images represent the reconstructed slices/phases, for instance, all the cardiac cine data.

The proposed architecture is depicted in Figure 1d and later denoted “simplified RAKI (SRAKI).” It can be compared to the RAKI model 1b, from which two layers and every nonlinear activation function were removed. As such, the proposed architecture is similar to a parallelized GRAPPA algorithm but with a different regularization, or simply consists of the residual path of the rRAKI approach. SRAKI will be trained in the same way as the other compared methods, that is, as in Figure 2a.

To avoid the extensive need for training of every cardiac phase followed by GRAPPA, RAKI, rRAKI, and SRAKI, illustrated in Figure 2a, we propose a strategy to make use of the temporal information redundancy between the different cardiac phases and the spatial information redundancy between adjacent slices. To visualize the impact of each acceleration component (cardiac phases, slices, or both), an ablation study was conducted, comparing training every phase and slice independently (SRAKI), multi‐phase (mpSRAKI), multi‐slice RAKI (msSRAKI), and the proposed method (combination of multi‐phase and multi‐slice).

For mpSRAKI, one random phase was selected for training, while all phases were reconstructed, as illustrated in Figure 2b. All slices were trained and reconstructed separately.

For msSRAKI, the middle slice of the stack of slices was used for training while all slices were reconstructed, and every phase was trained and reconstructed separately as depicted in Figure 2c. To facilitate the reading and comprehension, the notation of msSRAKI(A/B) is introduced, with B being the number of reconstructed slices based on the weights computed by the training of A number of slices; thus, when training the middle slice, the notation would be msSRAKI(1/9).

Training and reconstruction of the proposed model consisted of a combination of mpSRAKI and msSRAKI(1/3) (Figure 2d). The training is performed on the middle slice, while the reconstruction is done on all three slices, as we assumed the coil sensitivity maps to remain stable over only a limited region of space, whereas they should remain relatively constant throughout the cardiac cycle.

To confirm the difference in coil sensitivities between slices and phases, GRAPPA, SRAKI, and the proposed method weights from all subjects were extracted, and Pearson correlation coefficient was computed between all slices/phases and the middle slice/phase. Since GRAPPA weights are closely linked to SENSE coil sensitivities,^16^, ^17^ it can be assumed that the variation of GRAPPA weights would follow that of coil sensitivities.

The impact of the multi‐slice training strategy was also evaluated by varying the number of slices among the stack from training on a single slice for the whole stack, denoted msSRAKI(1/9); training on two slices from the stack with a 5 by 5 shift strategy (training on a slice and applying it on the two adjacent slices on each side) (msSRAKI[1/5]), training on three slices from the stack (training on a slice and applying it on the adjacent slice on each side) (msSRAKI[1/3]), and finally training with all nine slices from the stack, either training an individual model for each slice (msSRAKI[1/1]) or training a single model for all slices (msSRAKI[9/9]).

### 2.4 Quality metrics and statistic tests

To assess the different techniques, standard image quality metrics such as the peak signal to noise ratio (PSNR), the structural similarity index (SSIM)^18^ and the normalized mean square error (NMSE) were computed thanks to the available ground‐truth (GT) image reconstructed from fully sampled data, that served as the reference. The different metrics were computed as follows:

(1) $$PSNR=10\log\frac{MAX_{I}^{2}}{MSE}$$

where $MSE=\frac{1}{N}\sum_{i}^{N}{\left(I_{i}-Y_{i}\right)}^{2}$, I and Y are the reference and the predicted images, respectively, and $MAX_{I}$ the maximum pixel value of I.

(2) $$NMSE=\frac{MSE}{\frac{1}{N}\sum_{i}^{N}I_{i}^{2}}$$

with MSE the mean squared error between an estimation and a reference (in our case, the reconstructed image and the GT).

(3) $$SSIM\left(x,y\right)=\frac{\left(2\mu_{x}\mu_{y}+c_{1}\right)\left(2\sigma_{x}\sigma_{y}+c_{2}\right)\left(cov_{xy}+c_{3}\right)}{\left(\mu_{x}^{2}+\mu_{y}^{2}+c_{1}\right)\left(\sigma_{x}^{2}+\sigma_{y}^{2}+c_{2}\right)\left(\sigma_{x}\sigma_{y}+c_{3}\right)}$$

with μ the mean, $\sigma^{2}$ the variance and cov the covariance of x and y, the two images we want to measure the structural similarity of, that is, the reference (GT) and the reconstructed images. Variables $c_{1}$, $c_{2}$, and $c_{3}$ are defined in ref. [18].

The metrics were computed on a region of interest (ROI) manually defined encompassing the heart region, with a size of around 70 × 70 pixels wide. The ROIs were also used to display a zoom on the reconstructed images in part 3.

The reconstruction time for one frame (i.e., one cardiac phase) was also assessed and comprised the data loading time, training time, evaluation time, and reconstruction time (coil combination and inverse Fourier transform). These specific parts were timed independently with Python time module and summed at the end of the reconstruction.

Statistical tests were conducted to assess the statistical difference between two methods. A normality test (with the Shapiro test) was conducted to check the normality of all distributions. As none of them were normally distributed, a Wilcoxon test was later performed to determine the statistically significant difference between two methods.

In order to evaluate the influence of the slice position and cardiac phase on the kernel coefficients, the evolution of the weights between slices and phases was estimated for GRAPPA, SRAKI, and the proposed method. Pearson correlation coefficient was computed as such:

(4) $$\rho_{X,Y}=\frac{|\mathrm{Cov}\left(X,\bar{Y}\right)|}{\sigma_{X}\sigma_{Y}}$$

where *X* is the weight matrix reference (middle slice and middle phase), *Y* is the weight matrix from a different phase/slice, Cov is the covariance, σ the standard deviation. A $\rho_{X,Y}$ getting close to 1 will indicate that the weights are not affected by the phase or slice position.

### 2.5 Implementation details

All RAKI methods were implemented using PyTorch 1.13.1 and Python 3.8.8, with CUDA 11.6 and CuDNN 8.0, and ran on a server with one NVIDIA A100 GPU (40 GB). Training of the CNN weights was performed by optimizing the mean‐absolute error (MAE) loss function over 600 epochs (with ADAM optimizer, lr = 0.01 with a reduce learning rate on plateau scheduler [factor = 0.1, patience = 10 and threshold = 0.001]).

GRAPPA reconstructions were computed with the Pygrappa Python package, fixed with a kernel [5 × 5]. It should be noted that this implementation was not parallelized.

All statistical tests were computed on RStudio. The Python code of the algorithm used is available at: https://github.com/IADI‐Nancy/multi‐phase_SRAKI.

## 3 RESULTS

**FIGURE 3:**
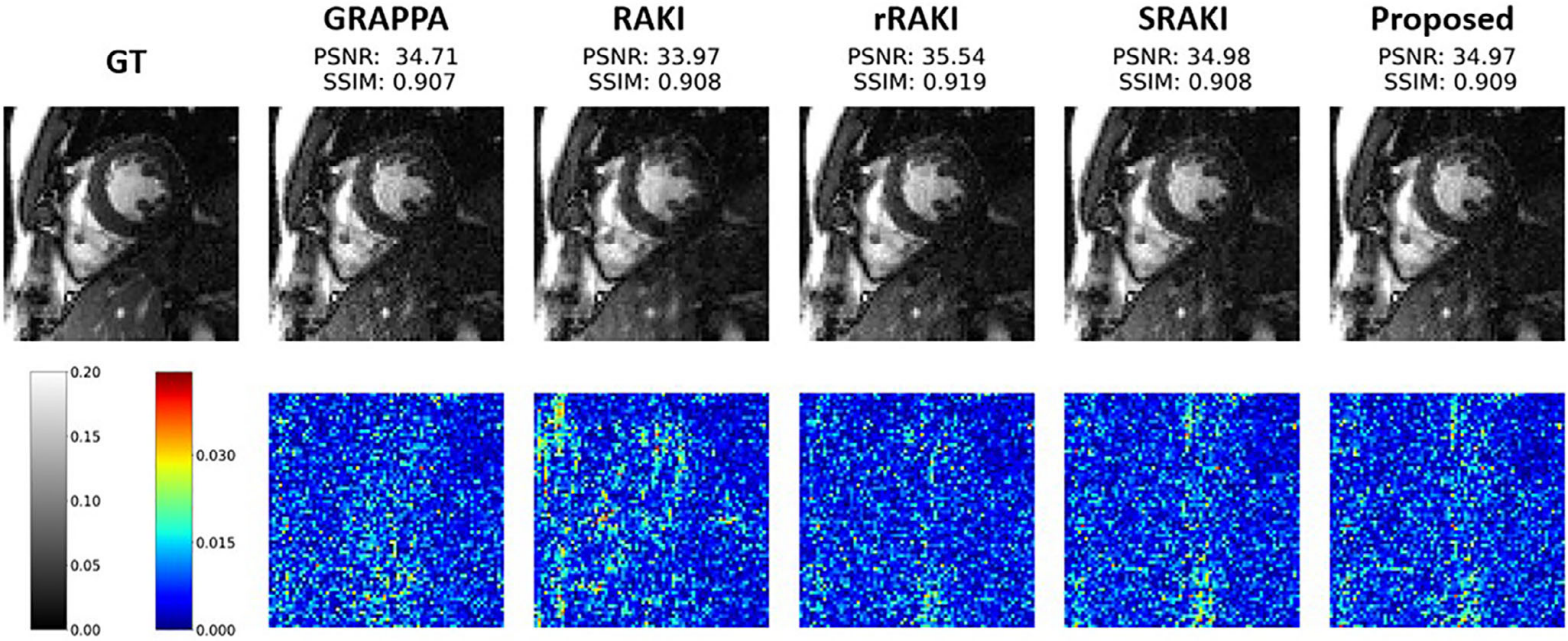
Reconstructed images for GRAPPA, RAKI, rRAKI, SRAKI, and the proposed method for R=4. Top row shows the magnitude reconstructed images for one patient and the second row the error maps for all methods. Both PSNR and SSIM for all methods are shown above the images.

**FIGURE 4:**
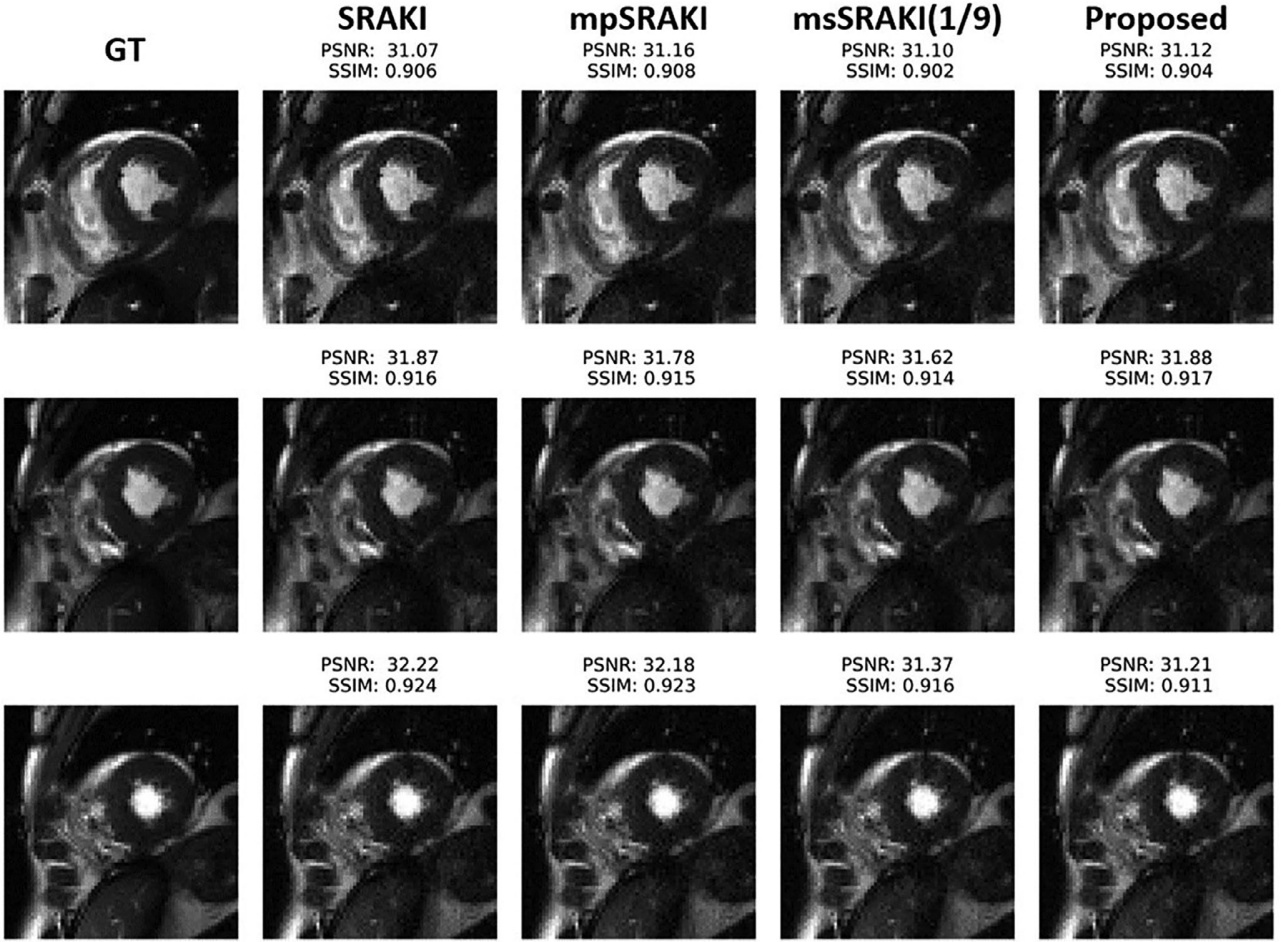
Reconstructed images for SRAKI, mpSRAKI, msSRAKI(1/9) and the proposed method at R=4. Middle row shows the trained slice while top and bottom rows show the adjacent and untrained slices. Both PSNR and SSIM for all methods are shown above the images.

All 2070 images (10 subjects, 9 slices, and 23 cardiac phases on average) were successfully reconstructed. Residual artifacts remained visible for a couple of reconstructions and consisted of darker bands across the images. Examples of reconstructions at R=4 are depicted in Figures 3 and 4. The results of image quality metrics and reconstruction time of the different methods for all undersampling rates are depicted in Table 1, while reconstructed images for different acceleration rates are available as in Supplementary Materials. There is a clear decrease of performance with the increase of the undersampling rate.

**TABLE 1:** Metrics and reconstruction time results at different undersampling rates R for GRAPPA, RAKI, rRAKI, SRAKI, mpSRAKI, msSRAKI(1/9), and the proposed method.

*R* = 4	PSNR↑	SSIM↑	NMSE↓	Time (s)↓
GRAPPA	34.03±3.69	0.909±0.035	0.0103±0.0065	3.00±0.473
RAKI	32.25±2.73	0.899±0.031	0.0148±0.0118	2.285±0.230
rRAKI	34.08±3.25	0.916±0.029	0.0098±0.0059	2.861±0.269
SRAKI	34.05±3.20	0.915±0.029	0.0098±0.0052	1.985±0.195
mpSRAKI	34.09±3.19	0.915±0.030	0.0097±0.0049	0.110±0.015
msSRAKI(1/9)	30.71±4.34	0.856±0.079	0.0295±0.0431	0.225±0.028
Proposed	33.21±3.01	0.904±0.032	0.0118±0.0072	0.062±0.010

*R* **= 5**	PSNR↑	SSIM↑	NMSE↓	Time (s)↓
GRAPPA	31.41±3.66	0.859±0.048	0.0193±0.0148	2.766±0.714
RAKI	31.22±3.11	0.876±0.040	0.0198±0.0187	5.247±3.730
rRAKI	32.03±3.29	0.883±0.038	0.0165±0.0139	2.767±0.276
SRAKI	31.93±3.29	0.881±0.39	0.0167±0.0130	1.968±0.310
mpSRAKI	31.94±3.28	0.881±0.038	0.0165±0.0109	0.109±0.021
msSRAKI(1/9)	29.19±4.08	0.825±0.084	0.0401±0.0575	0.205±0.032
Proposed	31.31±2.95	0.871±0.040	0.0186±0.0112	0.058±0.010

*R* **= 6**	PSNR↑	SSIM↑	NMSE↓	Time (s)↓
GRAPPA	30.15±3.09	0.844±0.069	0.0246±0.0170	2.515±0.425
RAKI	30.80±2.84	0.872±0.036	0.0210±0.0157	2.167±0.214
rRAKI	31.06±2.93	0.873±0.040	0.0201±0.0163	2.815±0.248
SRAKI	30.46±2.90	0.871±0.041	0.0229±0.0172	1.574±0.190
mpSRAKI	30.46±2.94	0.871±0.040	0.030±0.0173	0.0912±0.013
msSRAKI(1/9)	29.06±3.50	0.837±0.072	0.0367±0.0380	0.175±0.019
Proposed	30.29±2.87	0.868±0.044	0.0242±0.0195	0.054±0.017

*R* = 8	PSNR↑	SSIM↑	NMSE↓	Time (s)↓
GRAPPA	29.24±3.29	0.859±0.045	0.0320±0.0245	3.020±0.659
RAKI	29.28±2.82	0.860±0.040	0.0299±0.020	2.298±0.243
rRAKI	29.20±2.87	0.864±0.041	0.0308±0.0210	2.903±0.290
SRAKI	28.58±3.14	0.862±0.042	0.0369±0.0268	1.695±0.234
mpSRAKI	28.61±3.14	0.863±0.042	0.0366±0.0267	0.101±0.015
msSRAKI(1/9)	28.03±3.25	0.842±0.057	0.0429±0.0323	0.194±0.026
Proposed	28.51±3.10	0.860±0.043	0.0374±0.0261	0.054±0.009

Abbreviations: GRAPPA, generalized autocalibrating partially parallel acquisitions; NMSE, normalized mean square error; PSNR, peak signal to noise ratio; RAKI, robust artificial neural networks for k‐space interpolation; rRAKI, residual RAKI; SRAKI, simplified RAKI; SSIM, structural similarity index.

On average, the reconstruction time for mp‐msSRAKI(1/3) was divided between 21 ms of data loading, 28 ms for the training and inference, and 5 ms for the reconstruction.

### 3.1 Comparison methods

**FIGURE 5:**
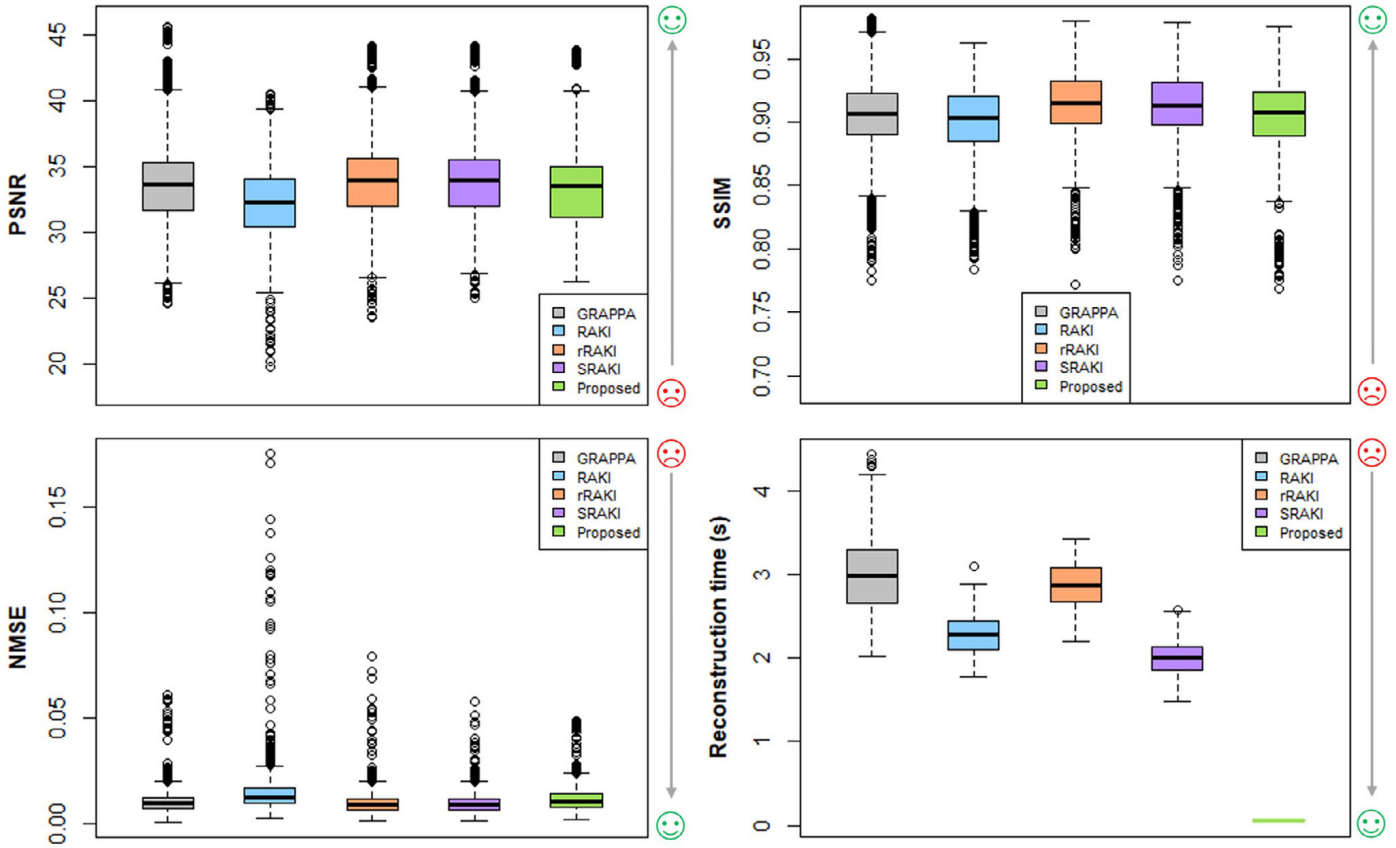
Boxplots of quality metrics and reconstruction time for all methods at R=4: GRAPPA, RAKI, rRAKI, SRAKI and the proposed architecture.

Boxplots of the PSNR, SSIM, NMSE, and the reconstruction time for GRAPPA, RAKI, rRAKI, sRAKI, and the proposed method for R=4 are illustrated in Figure 5. SRAKI and rRAKI seem to yield similar results, which is confirmed by Table 1 at R=4 (PSNR ≈34.07±3.20). RAKI has significantly worse results than all methods at R=4 (PSNR =32.25±2.73), while GRAPPA ranks close to the other two with PSNR ≈34.03±3.69.

Our proposed and simplified method seems to have slightly poorer quantitative results than rRAKI and SRAKI implementations (PSNR = 33.21 ± 3.01), while being significantly faster with an average reconstruction time of 0.062 ± 0.01 s. The proposed method is thus 48, 37, 46, and 32 times faster than GRAPPA, RAKI, rRAKI, and SRAKI, respectively.

For R=4 and R=5, we can see that rRAKI, SRAKI, and mpSRAKI yield similar as well as the best results. However, from *R* = 6, the results of SRAKI and mpSRAKI seem to decrease compared to rRAKI and GRAPPA, while RAKI seems to slightly outperform the other methods.

Reconstructed images without residual artifacts and their error maps for one patient and one cardiac phase for all comparison methods and the ground truth for R=4 are depicted in Figure 3, with a zoom on the heart region also used for computing quality metrics.

### 3.2 Ablation study

**FIGURE 6:**
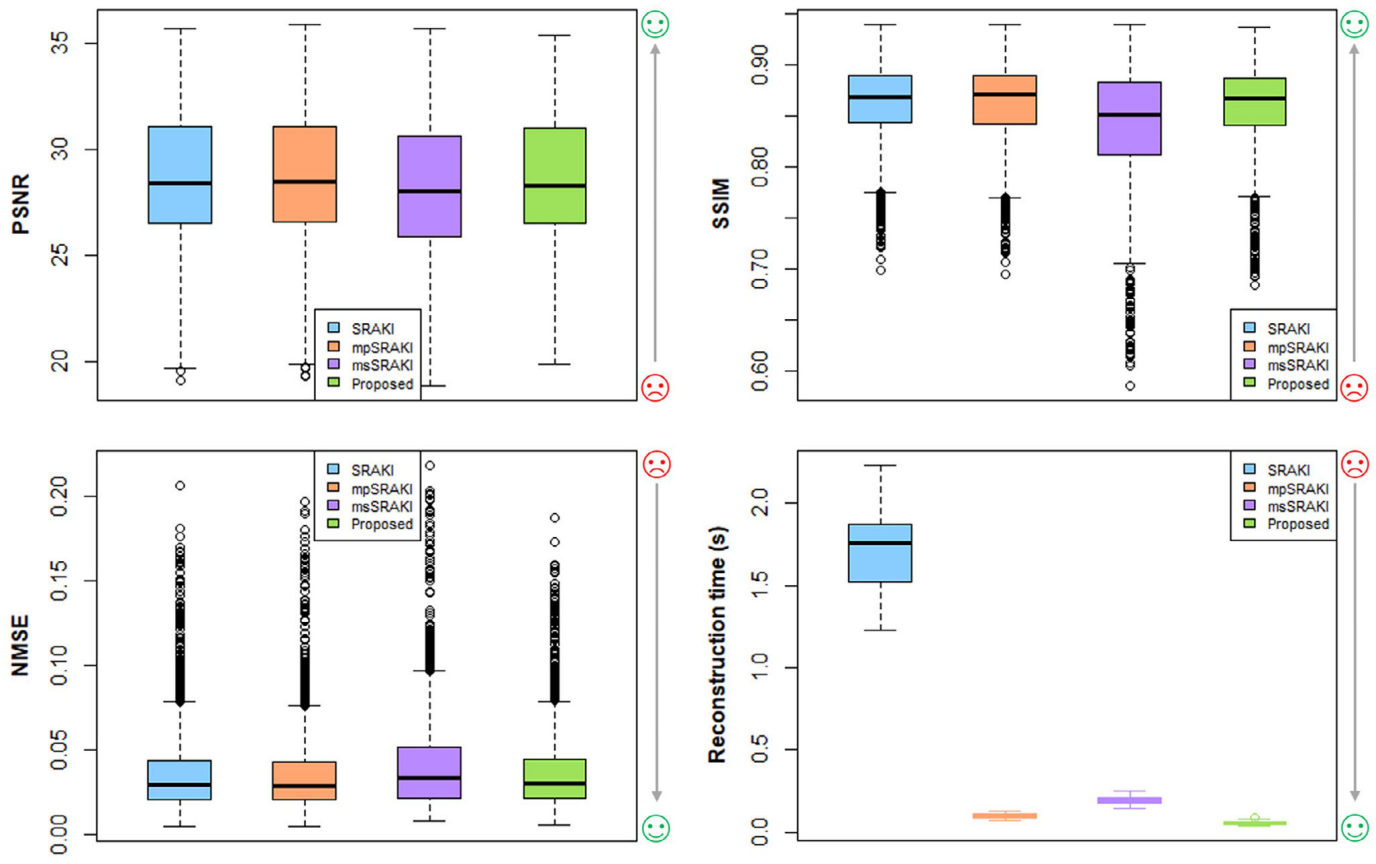
Boxplots of quality metrics and reconstruction time for all methods: SRAKI, mpSRAKI, msSRAKI(1/9) and the proposed method at R=4.

Figure 6 shows the boxplots of the different metrics computed on an ROI around the heart for R=4. SRAKI and mpSRAKI have no statistical difference for all quality metrics (SSIM ≈ 0.915 ± 0.030), while all the other methods are statistically different. The proposed method seems slightly poorer than SRAKI (SSIM = 0.904 ± 0.032). Only msSRAKI(1/9) boxplots show a huge lower performance for all metrics (SSIM = 0.856 ± 0.079), as well as a higher variability and a higher reconstruction time than mpSRAKI and the proposed method. The proposed method is 1.8 and 3.6 times faster than mpSRAKI and msSRAKI(1/9), respectively.

Reconstructed images of three adjacent slices at a fixed cardiac phase for the ablation study methods and the ground truth are depicted at R=4 in Figure 4, with a zoom on the heart region.

**FIGURE 7:**
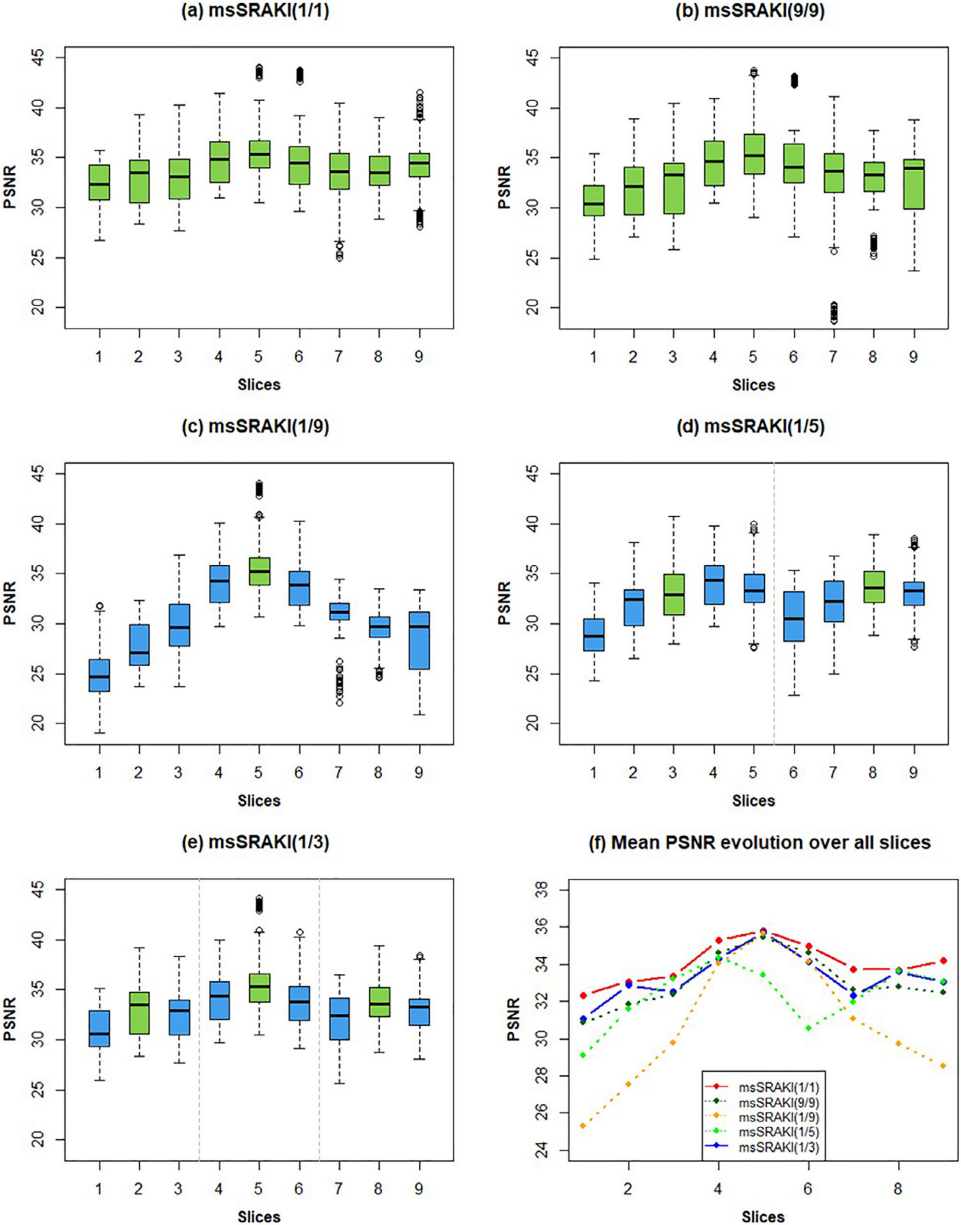
Boxplots of the PSNR for methods used in determining the optimal training slice: (a) msSRAKI(1/1), (b) msSRAKI(9/9), (c) msSRAKI(1/9), (d) msSRAKI(1/5), and (e) msSRAKI(1/3) at R=4. Green boxplots show the trained and reconstructed slices, while blue boxplots are only reconstructed slices. The dashed lines separate slices that were reconstructed based on the same weights (trained on the slice in the green boxplot(s)). The last graph (f) represents the evolution of the mean of the PSNR over all slices for the five methods.

To determine the impact of the training slice and the reconstruction performance, we tested and compared different training strategies: msSRAKI(1/1), msSRAKI(9/9), msSRAKI(1/9), msSRAKI(1/5), and msSRAKI(1/3) at R=4 (Figure 7). Boxplots and PSNR slope of the proposed method seemed to be the closest to the reference (msSRAKI[1/1]). Both msSRAKI(1/9) and msSRAKI(1/5) seemed to show significant PSNR decrease with the increasing distance between the training slice and the reconstructed slice. It can also be noted that for msSRAKI(9/9) network, which considers all slices for the training, the PSNR is a little higher for the middle slices compared to msSRAKI(1/3), while scores significantly drop for basal or apical slices (e.g., Slices 2, 8, and 9). This could mean that, although training has seen all slices, the weights are averaged and create generalization issues for some basal or apical slices.

**FIGURE 8:**
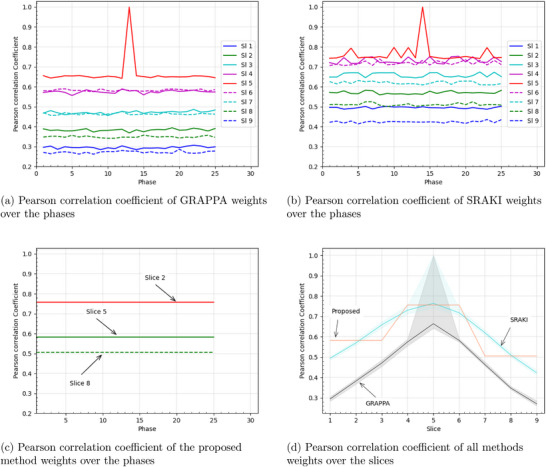
Pearson correlation coefficient of different methods weights between a chosen reference at phase = 13/slice = 5 (of GRAPPA weights for a and GRAPPA curve in d and of SRAKI weights for b, c, and SRAKI and Proposed curves in d) and all slices and phases. The evolution of the weights correlation coefficient is represented over the phases (a, b, and c) and the slices (d). The point in graphs where the coefficient is equal to 1 corresponds to the reference phase/slice. In a, b and c, the colors of the slices are the same for slices with the same distance to the reference slice of 5.

In order to confirm the assumption that coil sensitivities differ from one slice to another but stay relatively similar from one cardiac phase to the next, GRAPPA, SRAKI, and the proposed technique's weight correlation coefficients were computed. These correlation coefficient for a given patient are depicted in Figure 8.

Figure 8a seems to show that the correlation coefficient for GRAPPA, SRAKI, and the proposed technique remains constant throughout the cardiac cycle (except for the reference phase and slice where it is equal to 1). The evolution of the correlation coefficient with the slices can also be seen in these plots, where the furthest slices (in blue) have the lowest correlation coefficient. It is confirmed by the slope in Figure 8d where the correlation coefficient decays almost linearly with the distance to the reference slice. For the proposed method (Figure 8c), since only three trainings on different slices were performed, the correlation of those three slices seems on average similar to the correlation of the same slices for SRAKI. As for GRAPPA, the correlation coefficient for SRAKI decays with the distance to the middle slice, albeit to a lesser extent.

## 4 DISCUSSION

As can be seen on Figure 6, the proposed method is significantly faster than all comparison methods. It should, however, be noted that the Pygrappa implementation could probably be optimized as it consists of a non parallelized implementation. The reconstruction time could be further reduced when using parallelization ^19^ or even GPU‐optimized RAKI implementations.^14^ This study demonstrated that training the SRAKI model on the ACS data from a single cardiac phase and on the central slice of three adjacent slices allows for the reconstruction of high‐quality images. However, the study was limited to data with retrospective acceleration; further study is therefore required to develop and optimize an acquisition strategy where only ACS data from a single cardiac phase and a single slice is acquired, which would also considerably reduce the acquisition time.

Furthermore, our results seem to indicate that SRAKI architecture allows for good image reconstruction. The use of a more complex architecture, with multiple layers and nonlinear activation units, does not seem to be necessary for cardiac cine applications at low undersampling rates (R=4 or R=5). The introduction of residual RAKI has also showcased similar behavior depending on the final applications, with performance of RAKI and rRAKI being slightly different for brain or knee images.^15^ One promise of the newly released residual RAKI was to reduce some artifacts seen in RAKI by adding a linear network to guide the training of the nonlinear CNN. We seemed to demonstrate that on cardiac cine data, except for higher undersampling rates (R>6), one can go even further by simplifying the network (since SRAKI is similar to the linear path of rRAKI), which seems to perform as well as both RAKI and rRAKI while being significantly faster.

The reconstructed images from SRAKI and mpSRAKI on Figure 4 shows almost no visible difference, while some artifacts can be detected with the msSRAKI(1/9) and mp‐msSRAKI(1/3). The best trade‐off between imaging quality and reconstruction time would need to be considered specifically for each application and its limits. As it is, both mpSRAKI and mp‐msSRAKI(1/3) seem to present sufficient performances for this purpose, with mpSRAKI providing better imaging quality and mp‐msSRAKI(1/3) better time efficiency. The choice between the methods would also depend on hardware performance. Noise structures seemed to be different for the different reconstruction techniques, with GRAPPA seemingly inducing more diffused noise while RAKI‐based methods appeared to generate more structured artifacts on the images. The proposed technique, which is similar to the GRAPPA architecture, instead follows the noise structure of RAKI‐based reconstructions, pointing out the potential effect of the regularization and optimization technique.

The correlation coefficients between GRAPPA, SRAKI, and the proposed technique kernel weights of different phases and slices indicate that, as we assumed, those weights do not vary much throughout the cardiac cycle but evolve rapidly and linearly with the distance between the slices. Those weights are highly dependent on coil sensitivities and therefore the geometry of the coils. The slopes, corresponding to the evolution of kernel weights in Figure 8d, seem to follow the PSNR slope of the msSRAKI(1/9) method in Figure 7.

Given that the proposed RAKI‐based techniques are scan‐specific, requiring to be trained from scratch for each new acquisition, one assumes that they generalize as well as GRAPPA and that similar performance would be obtained from data acquired with different cardiac views, on scanners from different vendors, field strengths, or with different pulse sequence parameters. Similarly, as RAKI has previously been applied to 3D data with convincing results,^20^ the proposed algorithm could also be extended to 3D datasets. Furthermore, it should be noted that this model does not deal with motion; thus, it would not be efficient for free‐breathing data unless significant change is made to the implementation. For instance, respiratory binning could be implemented in order to avoid major respiration motion artifacts.

The proposed technique consists of a k‐space DL reconstruction approach, which offers several advantages, namely being a single‐shot learning technique that does not require a huge database but also works directly in k‐space, therefore eliminating the need to apply several back and forth Fourier transforms, as opposed to what has been proposed in GRAPPANet,^21^ with a k‐space UNet and an image‐space UNet, each minimizing a combination of SSIM and L1 loss. However, as the proposed technique is solely k‐space‐based, one noticed that some reconstructed images may contain “striping” artifacts. One investigated the use of k‐space‐specific loss functions, as several have already been suggested in the literature.^22^, ^23^, ^24^ Those loss functions account for the fact that energy is concentrated in the center of the k‐space, and that reconstruction of fine details and structures requires good interpolation in the outer space. Another possibility would be to associate an image‐based loss function, such as total variation, but that would require regular applications of a Fourier transform and would have a significant impact on the reconstruction time.

Implicit neural representation is an emerging technique that is gaining popularity in the biomedical image analysis field, as well as in k‐space‐based MRI image reconstruction.^22^, ^25^, ^26^ This technique aims at learning a continuous function of spatial coordinates representing the inherent image (or k‐space) and has already been suggested for scan‐specific reconstruction methods. Presented results seem quite promising, with few reconstruction artifacts.^27^ This approach might be interesting for cardiac cine applications and should be explored in further works.

## 5 CONCLUSION

In this study, we proposed a novel method designed to accelerate RAKI‐based image reconstruction for cardiac cine applications. In particular, we considered the use of a simplified architecture with restricted number of cardiac phases and slices on which the model is trained. By taking into consideration the small variations between adjacent slices and cardiac phases, the training time and the overall reconstruction time were significantly reduced. The method brought comparable results and image quality to other standard reconstruction techniques when computed on multi‐slice fully sampled data from the OCMR database.

## CONFLICT OF INTEREST STATEMENT

The authors have no relevant conflicts of interest to disclose.

## Supporting information

Supporting Information

Supplemental Video 1

Supplemental Video 2
