# Supplementary material for: Accelerated RAKI reconstruction for multi‐slice cardiac cine applications
Source: Med Phys. 2025 Nov 19;52(12):e70145. doi: 10.1002/mp.70145 (PMC12630071; doi:10.1002/mp.70145)
Supplement: Supplementary file 1 — Supporting Information [file MP-52-0-s003.pdf]

# Supplementary Materials

Reconstructed cine images of the middle slice for both the ground truth and mpSRAKI are provided. Reconstructed images of the mp-msSRAKI(1/3), mpSRAKI, GRAPPA and the ground truth are available for different acceleration rates ($R = [4, 5, 6, 8]$).

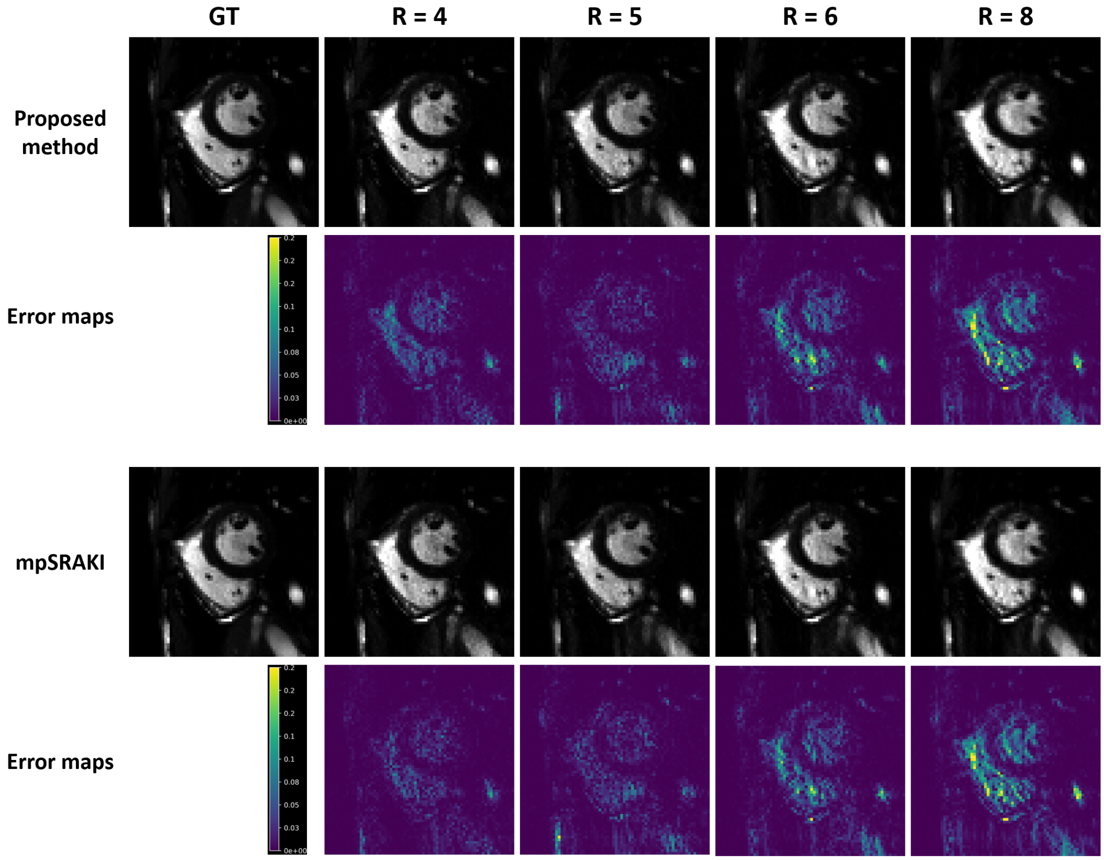


Supplementary figure: Reconstructed images for the proposed method and mpSRAKI at different acceleration rates ($R = [4, 5, 6, 8]$) with the corresponding error maps, compared to the GT.
